# Planning for Supportive Green Spaces in the Winter City of China: Linking Exercise of Elderly Residents and Exercise Prescription for Cardiovascular Health

**DOI:** 10.3390/ijerph17165762

**Published:** 2020-08-10

**Authors:** Hong Leng, Shuyuan Li, Huimin Zhao, Yan Song, Qing Yuan

**Affiliations:** 1 School of Architecture, Harbin Institute of Technology, Harbin 150001, China; hitlaura@126.com (H.L.); lishuyuan@hit.edu.cn (S.L.); hitzhuimin@163.com (H.Z.); 2 Key Laboratory of Cold Region Urban and Rural Human Settlement Environment Science and Technology, Ministry of Industry and Information Technology, Harbin 150001, China; 3 Department of City and Regional Planning, University of North Carolina at Chapel Hill, Chapel Hill, NC 27599, USA; ys@email.unc.edu

**Keywords:** winter city, green space, cardiovascular health, exercise prescription, exercise characteristics, the elderly

## Abstract

The elderly population have a high incidence of cardiovascular disease and are the main users of green spaces, such as city parks. Creating supportive green spaces for exercise for the elderly is of great significance to promote their cardiovascular health. The winter cities have a severely cold climate and high incidence of cardiovascular disease, while the elderly, especially those with cardiovascular disease, face more challenges when participating in exercise in the green spaces. In the context of the winter cities, the kinds of exercise the elderly participate in are more conducive to their cardiovascular health, and determining the factors of the green spaces that are supportive for exercise for cardiovascular health in the winter are of particular interest. Taking Harbin, a typical winter city in China, as an example, this study aims to identify the exercise characteristics of elderly residents in the green spaces in winter, to link them with the principles and contents of exercise prescription for cardiovascular health, to identify the deficient factors of the green spaces in supporting exercise for cardiovascular health, and to put forward optimization design implications. Mixed qualitative methods including interviews, a questionnaire, and field observation were used to identify special behavioral characteristics and spatial factors involving winter exercise in the green spaces among the elderly. The results showed that: (1) about 42.4% of the participants had a gap with the principles of exercise prescription for cardiovascular health. Their exercise items were generally consistent with the principle of low-intensity exercise, but some of them had the problems regarding early exercise time and insufficient exercise duration and frequency. (2) Insufficient supportive factors of the green spaces mainly included facilities allocation, comfort, safety, accessibility, and air quality. Facilities allocation involved walking paths, rehabilitation facilities, auxiliary facilities, and guidance facilities; comfort involved sunlight conditions of the exercise areas; safety involved slippery roads and sites with ice and snow and medical accidents; accessibility involved the proximity, the safety of connecting roads, and the movement of the elderly; air quality involved the planting of evergreen trees. Accordingly, the design implications were given in order to bridge the supportive gap of the green spaces for exercise for cardiovascular health in the elderly population.

## 1. Introduction

Cardiovascular disease is the world’s leading health killer. The main population of patients suffering the disease are the elderly. Medical studies show that the prevalence of hypertension, ischemic cardiovascular disease, atrial fibrillation, and coronary heart disease among the elderly is 2–4 times higher than that among the younger population [1,2]. China has become an aging society. By the end of 2017, the number of people over 60 years old was about 240 million, accounting for 17.3% of the total population of the country [3], and the prevalence of cardiovascular disease among the elderly residents was as high as 62% [4]. By 2050, the proportion of the elderly population is expected to reach 34.1% [5]. In the face of this major public health concern, which is also a serious threat to healthy aging, the prevention and treatment of cardiovascular disease is urgent.

The World Health Organization (WHO) points out that both environmental and behavioral factors play an important role in the prevention and treatment of cardiovascular disease. By creating a supportive environment for healthy behaviors and an active lifestyle, the risk disease can be reduced in a large population [6]. By promoting positive behavioral factors such as healthy diet, regular exercise, and non-smoking, most cardiovascular disease (such as 80% of premature heart attacks and strokes) can be prevented [7]. Therefore, according to the WHO, creating a supportive environment and advocating healthy behavior are the key measures that countries can implement to prevent and control cardiovascular disease.

In terms of environment, as the most important spatial carrier of exercise in the urban space [8], and having other advantages such as improving air quality, enhancing the social communication, and reducing pressure [9], green spaces have been widely found to be associated with cardiovascular health [10,11,12,13,14,15]. According to the U.S. National Environmental Protection Agency, green space is defined as land that is partly or completely covered with grass, trees, shrubs or other vegetation, including parks, squares, playgrounds, residential yards, schoolyards, public seating areas. and vacant lots, which can provide recreational areas for residents and help to enhance the beauty and environmental quality of neighborhoods [16].

In terms of behavior, exercise plays an important role in the prevention and treatment of cardiovascular disease. As the basis of energy balance and weight control, exercise can improve endothelial function, thus enhancing vasodilation and vasomotor function [17]; it also helps one to lose weight, control blood sugar, and improve blood pressure, blood fat, and insulin sensitivity [18,19]. In addition, the impact of exercise is natural. Taking its impact on the heart as an example, its stimulation on the heart is physiological and does not cause cell death, fibrosis, and heart function decline, so it is different from pathological heart reconstruction [20,21]. Therefore, in medical research, exercise is considered an essential, economic, and effective measure in the prevention and treatment of cardiovascular disease, which should be placed on the same important position as diet control [22,23,24].

Winter exercise plays an important role in the prevention and rehabilitation of cardiovascular disease in the elderly. Winter exercise can help promote blood circulation and improve vascular tolerance and keep its elasticity [25]. Compared with indoor exercise, outdoor exercise provides fresh air and helps enhance social interaction [26], which are not only important health needs of the elderly, but also beneficial factors to promote cardiovascular health overall [27]. Although the vasoconstriction and spasm caused by low temperature is dangerous for some patients with cardiovascular disease, such as heart disease [25], medical and sports scholars generally believe that the benefits gleaned from winter exercise far outweigh the risks [28,29]. China’s winter cities are areas with high incidences of cardiovascular disease due to the influence of the severely cold climate [30]. In winter, both the frequency and total amount of exercise of the elderly decrease [31], which has adverse effects on their cardiovascular health.

Providing supportive green spaces through reasonable planning and design is highly significant for encouraging the elderly to adhere to exercise and thus maintain their cardiovascular health in the winter. There are a large number of elderly residents in winter cities; different from those in the high-income group who can migrate to warmer areas during winter, the middle- and low-income elderly are more dependent on daily fitness resources. However, most of the winter cities in China have high-density human settlements, where the total amount of indoor exercise rooms is quite small. At the same time, due to factors including the proximity to nature, social interaction, and exercise cost, green spaces are important places for the elderly to exercise in winter. The severely cold climate also has an impact on the green space environment, leading to reduced safety and comfort [31], which hinders the elderly from exercising. Building a good green space environment will be conductive to providing a supportive environment for exercise, promoting the occurrence of daily exercise of the elderly in winter, and reducing the risk of cardiovascular disease to a certain extent.

There are a growing number of studies focusing on the impacts of green space on cardiovascular health [10,11,12,13,14,15]. The involving green space factors include the scale, layout, physical activity resources, and the use [10,11,12,13]. The involving cardiovascular health outcomes include obesity, hypertension, diabetes, and other risk factors, as well as the morbidity and mortality of cardiovascular disease such as stroke [10,11,14,15]. A review of 12 studies screened from North America, Europe, and Oceania found that most studies showed that residents in settlements with more green spaces had lower cardiovascular disease mortality [10]. Communities closer to green spaces had lower morbidity from diabetes and stroke [11]. Studies based on communities in the U.S. found that residents living in communities that provided 19% of exercise resources or 20% of walking environments were more likely to have ideal cardiovascular health scores [12]. Another study in Lithuania showed that the use of the green spaces was negatively associated with cardiometabolic risk, helping to reduce the risk of disease [13]. In addition, a number of forest medicine studies showed that walking in the forest was beneficial to cardiovascular health [32,33]. However, although the number of studies in this field have been increasing, few identify the exercise characteristics of the elderly in the context of winter cities, especially the elderly with cardiovascular disease, and few explore what exercise methods are conducive to elderly cardiovascular health and what factors of green spaces are more conducive to supporting exercise for cardiovascular health.

The effects of exercise are related to its item, intensity, duration and frequency, and different exercise methods should be adopted for people in different age groups and for different exercise purposes [34]. In the 1950s, American sports physiologist Kapovic put forward the concept of exercise prescription, which refers to “a scientific and quantitative periodic exercise plan for individual physical condition” [35]. In promoting cardiovascular health, exercise prescription has important value. Research shows that exercise prescription can reduce the blood pressure and blood sugar of patients with cardiovascular disease, and improve cardiopulmonary function, exercise endurance, and quality of life [36]. Through exercise prescription, the specific exercise methods that are beneficial to the cardiovascular health of the elderly can be understood, and by meeting the requirements of the exercise prescription for cardiovascular health, the elderly can obtain scientifically backed, safe, and effective exercise outcomes.

Based on mixed qualitative methods, the aim of this study was to identify the exercise characteristics of the elderly residents in green spaces in winter cities and link them with the principles of exercise prescription for cardiovascular health in order to identify the deficiency of green spaces in supporting exercise for cardiovascular health, and thus propose targeted design implications. In terms of study design, the previous studies on the green spaces and exercise and other leisure physical activities mostly adopted quantitative methods [37,38,39,40,41,42]. For example, a study in New Zealand using the multi-level model found that the total amount of physical activity was larger in greener communities [43]. Another study in England using logistic regression found that residents living closer to green spaces had higher levels of physical activity [44]. However, it should be noted that exercise behavior is not only influenced by objective indicators such as distance, mixed space layout, and so on, but also by the subjective perception of the environment; some factors of the green spaces cannot be fully measured by quantitative indicators [45,46]. Especially in the context of the winter cities, the factors of the green spaces in supporting elderly exercise may not be quantified precisely. Taking accessibility as an example, under the influence of a severely cold climate, the accessibility of the green spaces would involve not only the proximity on the physical dimension, but also the safety risk caused by snow and ice conditions of the connecting paths and the physical movements of the elderly, both of which are difficult to quantify. More importantly, different from typical research on exercise behavior relying on quantitative analysis, qualitative research offers unique opportunities for understanding complex and nuanced situations where interpersonal ambiguity and multiple interpretations exist, which could help identify special factors [47]. Therefore, we chose to use qualitative methods in our study. It should be pointed out that some elderly patients, such as those with heart disease, need to follow a more prudent exercise prescription advice given by doctors, especially those who may be unsuited to participate in outdoor exercise in winter. Therefore, our study mainly focuses on providing spatial support for the elderly who can participate in and be suitable for exercise in the green spaces.

## 2. Materials and Methods

### 2.1. Study Site

Harbin (44°04′ N–46°40′ N, 125°42′ E–130°10′ E), a typical winter city in China, was selected as the research city. Its climate is characterized by four distinct seasons, a long and cold winter, a short and hot summer, and short spring and autumn with rapidly changing temperature. Its annual average temperature is 3.6 °C. Winter starts from November and lasts until February of the following year, with an average temperature of −14.2°C.

At the end of 2016, the total population of Harbin was 9.621 million, accounting for 0.7% of the total population of China. Among them, 1.924 million were the elderly residents over 60 years old, accounting for 20.0% of the total population in the city [48]. Harbin consists of 9 administrative districts, among which Nangang District, Daoli District, Xiangfang District, and Daowai District are the four most important ones with relatively concentrated urban populations and the continuous development of urban construction.

The study sites were selected from the green spaces and hospitals distributed in the four main districts. In terms of the green spaces, according to the actual use of green spaces in different spatial forms, the park, the square, the playground, and the residential yard were identified as the research objects. On this basis, the typical green spaces that had high exercise utilization rate were selected as the field observation samples, including the Majiagou Park, the Beixiu Square, a playground, and a residential yard. In terms of the hospitals, according to the construction level of the Department of Cardiology and the number of patients, three hospitals with the key departments of Cardiology in Harbin were selected as the study sites.

### 2.2. Survey Methods

In order to comprehensively understand the exercise characteristics of the elderly residents in the green spaces, mixed survey methods were used, including interviews with doctors, a questionnaire given to the elderly participants, interviews with the elderly participants with cardiovascular disease, and field observation in the green spaces. The investigation includes two stages: pre-survey and formal survey. Prior to the formal survey, we conducted a pre-survey, including interviews with doctors and patients in the hospitals and a questionnaire survey with the elderly regarding green spaces. The questionnaire and interview outline were determined accordingly. On this basis, we conducted the formal questionnaire survey, interview survey, and field observation. The study was conducted in accordance with the Declaration of Helsinki, and the protocol was ethical reviewed by the School of Architecture, Harbin Institute of Technology. The implementation process and specific contents of each part were as follows.

#### 2.2.1. Interview Survey with the Doctors

The purpose of interview survey with doctors was to understand the principles and contents of exercise prescription for cardiovascular health, and to identify the requirements for green spaces in supporting exercise for cardiovascular health from the perspective of doctors. Before we conducted the interviews, we consulted correlative literature on exercise prescription for cardiovascular health [36,49,50,51], winter exercise [28,52,53,54], and winter cardiovascular health protection [55,56,57,58] as preparation. In February 2019, we interviewed three well-known experts in the field of cardiovascular disease prevention and control from three different hospitals in Harbin. The interview questions included: (1) what are the contents and principles of exercise prescription for the cardiovascular health of the elderly? and (2) what are the special requirements for the green spaces in supporting exercise for cardiovascular health?

#### 2.2.2. Questionnaire Survey with the Elderly in the Green Spaces

The pre-survey for the questionnaire was conducted in February 2019 in the green spaces, recruiting 52 elderly participants in total. Based on the pre-survey results, the questionnaire was revised and the final version determined, which consist of two parts. The first part collected basic personal information (age, gender, education level, and monthly income) about the participants. The second part covered the exercise characteristics of the elderly, including the exercise items, exercise time, exercise duration, exercise frequency, the total exercise amount per week, the factors hindering exercise persistently, the factors promoting exercise persistently in the green spaces, and the need for green spaces. From November to December 2019, the formal questionnaires were distributed in the four selected green spaces, with 230 copies distributed and 188 valid questionnaires recovered, with a recovery rate of 81.7%. Participants who met the following conditions were recruited: (1) living in the main urban areas of Harbin; (2) over 60 years old; (3) participating in exercise in the green spaces. We distributed small gifts including handkerchief papers and pens to the participants who filled in the questionnaire to improve their enthusiasm for cooperating with our survey and to express our gratitude for their participation.

#### 2.2.3. Interview Survey with the Elderly Patients with Cardiovascular Disease

The pre-survey for the interview was conducted in February 2019 in the outpatient and inpatient cardiology department of a well-known hospital in Harbin with 29 interviewees. The formal interview outline was then determined according to the pre-survey results. The formal interview survey was conducted in the outpatient and inpatient departments of three hospitals in December 2019. The interview for each patient was conducted under the condition that they knew the purpose and contents of the interview and participated voluntarily. Forty elderly patients participated in the formal interviews in total. The contents of formal interview included basic personal information (age, gender), sickness status, and the specific exercise characteristics in the green spaces.

#### 2.2.4. Field Observation in the Green Spaces

**Figure 1:**
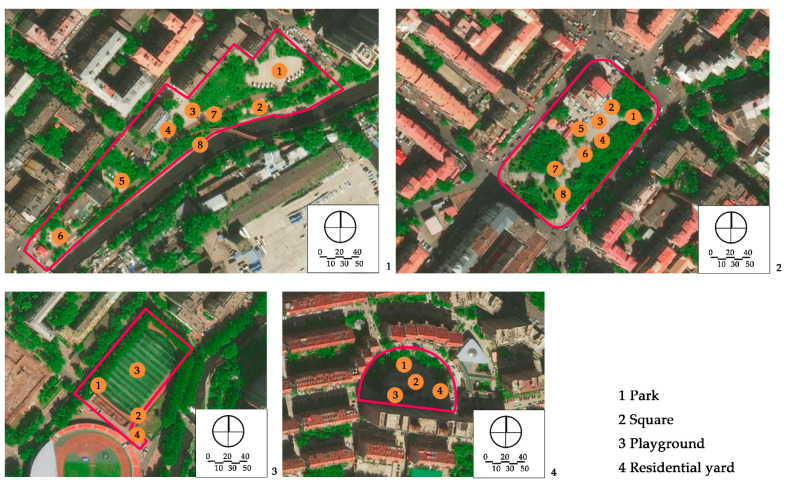
Distribution of the observation sampling sites in each green space.

A field observation was carried out in December 2019 to determine the actual exercise characteristics of the elderly in green spaces and the factors of green spaces supporting exercise for cardiovascular health, The distribution of observation sampling sites in each green space is shown in Figure 1. The methods of on-site recording and rapid photo taking were used to carry out statistics (the number of the exercisers participating in different exercise items at different time points in each green space) and spatial characteristics recorded from 6:00 to 20:00 at each whole time point. The investigation personnel conducted each round of observation according to a fixed route and stayed for 10 min in each sampling site.

### 2.3. Statistical Analysis

The analysis included two steps: (1) at the behavioral level, comparing the exercise characteristics obtained from questionnaire, interview, and field observation with the principles of exercise prescription for cardiovascular health obtained from interview with the doctors to find the characteristics that did not meet the requirements; (2) at the spatial level, in combination with the questionnaire, interview, field observation, and the compared results in step one, analyzing and depurating the insufficient factors of the green spaces in supporting exercise for cardiovascular health.

WPS Office Software Excel 2019 was used to conduct statistical processing and chart drawing of the data obtained from the questionnaire and the number of the seniors participating in different exercise items at different time points in each green space from the field observation. Baidu Map, CAD 2019 (Computer Aided Design) and Adobe Photoshop CS6 (Adobe, San Jose, CA, USA) were used to draw the distribution of observation sampling sites and exercise behavior maps of the green spaces.

## 3. Results

### 3.1. Participant Characteristics

The characteristics of the senior participants taken from the questionnaire and the interview survey are presented in Table 1. Overall, 230 participants participated in the questionnaire survey in the green spaces, of which 188 fully completed the questionnaire. Forty seniors participated in the interviews at the hospitals. The majority of participants were male, and the average age was 67.5 and 68.0 years old, respectively, for the questionnaire and interview. In the questionnaire survey, 72.0% of the participants had an education level less than or equal to high school and 78.8% of them earned a monthly income less than 5000 yuan, which put them at a low-to-middle income level according to the classification standards for different income groups from the National Bureau of Statistics of China (Table A1).

### 3.2. Exercise Characteristics and Exercise Prescription for Cardiovascular Health

#### 3.2.1. Principles and Contents of Exercise Prescription for Cardiovascular Health

According to the results of the interviews with the doctors, the exercise prescription for cardiovascular health includes five elements: the exercise item, the exercise time, the exercise duration, the exercise frequency, and the precautions. Compared with the general exercise prescription, its special guiding principles includes: the exercise item should be low-intensity rather than high-intensity exercise, the exercise time should not be in the early morning, and the total amount of exercise for the elderly per week should be at least >3 times a week and >30 min each time [50,59]. Based on these principles, the formulation of exercise prescription could be individualized according to patients’ health status assessment and risk stratification.

In terms of the exercise item, the elderly are recommended to do low-intensity exercise, and the items that were most conducive to cardiovascular health include swing exercise, aerobic exercise, walking, and Tai Chi [60]. Among them, walking, as the most safe, simple, and easy to adhere to exercise, is considered to be the first choice for elderly diabetics. Tai Chi, which helps to control weight, reduce heart rate, improve metabolism, and cardiopulmonary function, is safe, acceptable, and feasible even for the elderly patients with cardiovascular disease who are very weak [61]. In terms of exercise time, outdoor exercise time in winter should not be in the early morning when the low temperature would cause vasoconstriction and spasm, while an appropriate temperature is conducive to reduce the impact. In terms of exercise duration and frequency, the exercise needs to maintain a certain cycle and frequency. If exercise is stopped for 3 days or more, the accumulated health benefits such as improved insulin sensitivity will disappear. Therefore, the exercise should be implemented persistently.

#### 3.2.2. Exercise Items and Prescription Principles

The seniors participated in various activities such as walking, using exercise equipment, Tai Chi, dancing, and so on according to their interests, habits, and physical conditions. Walking was the main exercise item of the participants, followed by exercise with equipment and jogging. The results of the exercise items of the participants in the formal questionnaire survey are shown in Table 2. The interview results revealed that walking and stretching were the main exercise items of the elderly patients with cardiovascular disease. Special rehabilitation exercise items included stretching with rehabilitation equipment and walking with handrails. In the field observation, we found that the elderly usually walked slowly; stroke patients usually had slower, smaller, and more unstable gait, and walking was their main exercise item. In general, the exercise items they participated in were consistent with the low-intensity exercise items in the principles of the exercise prescription for cardiovascular health.

#### 3.2.3. Exercise Time and Prescription Principles

The exercise times of the participants in the questionnaire survey are shown in Table 3; it can be interpreted that 9:00–11:00 was the most important time for the participants to exercise. The exercise time of the participants in different exercise duration groups are reported in Table 4 and shows that the majority of the participants who exercised for longer periods of time exercised in the green spaces at 9:00–14:00. However, 10.6% of the participants said that they were used to exercising from 6:00 to 8:00. The interview and behavior observation also found that some participants chose to exercise in the early morning, and some interviewees said that the air quality in the early morning was better. This was in conflict with the prescription principle of avoiding exercise early in the morning, which contains a certain risk for cardiovascular health in winter.

#### 3.2.4. Exercise Duration, Frequency, and Prescription Principles

According to the questionnaire survey, the daily exercise duration of most of the participants lasted 1 to 2 h (29.3%), but 39.9% of the participants had an exercise duration of 30 min or less. Most of the participants exercised in the green spaces 5 to 7 times a week (60.7%), but about 28.5% of the participants exercised less than 3 times a week (Table 5). According to the interview with the elderly patients, the daily exercise duration of most of the participants lasted 20 to 30 min. The exercise frequency of most of the participants was less than 3 times, followed by 3 to 5 times. Compared with the recommended total exercise amount per week (more than 3 times per week and more than 30 min per time) in the prescription principles, about 42.4% of the participants in the questionnaire survey had an insufficient total amount of exercise. In addition, the participants who had longer exercise duration averagely had higher frequency according to the cross-tab statistical results of exercise duration and exercise frequency (Table 6).

### 3.3. Green Space Factors in Supporting Exercise for Cardiovascular Health 

The area of the green spaces is reported in Table 7. Among them, the area of the residential yard accounted for approximately 5.17% of that of the residential district. The tree species in the green spaces included evergreen trees, deciduous trees, and shrubs, and among them the evergreen trees were mainly pine trees; the height of the evergreen trees was 10–20 m, the height of deciduous trees was 6–30 m, and the height of shrubs was 0.5–6 m. The tree species in the park, the square, and the residential yard included evergreen trees, deciduous trees, and shrubs, and the latter two were the main tree species. The tree species in the playground were mainly deciduous trees.

#### 3.3.1. Factors in Supporting Exercise Items 

The interview with the doctors and the field observation showed that each spatial form of the green spaces can support various types of exercise items beneficial to cardiovascular health. Different exercise items were supported by different spatial forms of the sites in the green spaces (Table 8). The flat paths supporting walking (the main exercise item) were the sites used by the participants most. There existed a mutual interference between the slow-moving people and fast-moving people, but no speed differentiation was marked in the existing green spaces; the space of the square was fuzzy, and there existed a certain streamline interference between the exercisers and passing people. In addition, there were no rehabilitation trails with handrails or any facilities with guidance on scientific exercise in the green spaces.

In the process of participating in the exercise, the participants warmed up, rested, and stretched in different stages, and used the seats, railings, and other facilities in the green spaces. Due to the severely cold climate, many participants brought cushions with them, but there were few hanging facilities in the green spaces. In addition, we found that some participants brought their own brooms, shovels, and other tools to clear the snow for the exercise sites; even if it snowed, they exercised actively.

#### 3.3.2. Factors in Supporting Exercise Time

**Figure 2:**
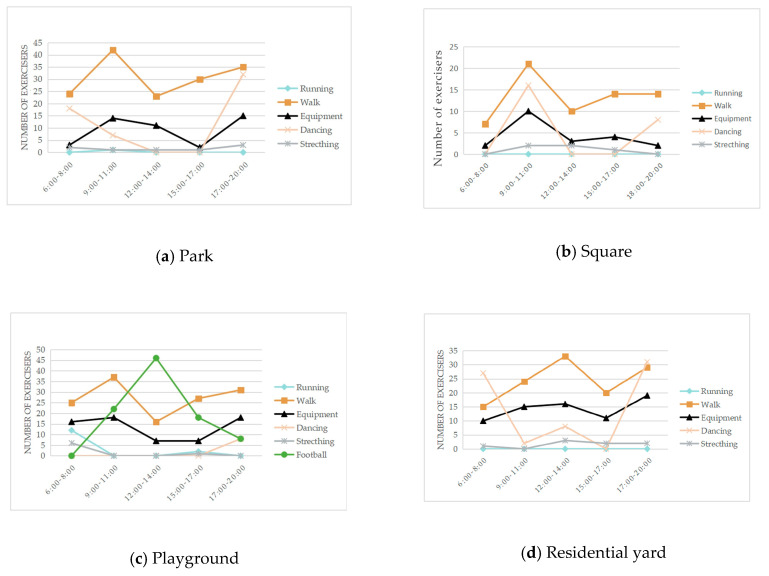
Number of exercisers at different time in the green spaces. (**a**) Number of exercisers at different time in the park; (**b**) number of exercisers at different time in the square; (**c**) number of exercisers at different time in the playground; (**d**) number of exercisers at different time in the residential yard.

The number of participants participating in different exercise items at different time points in each green space obtained from field observation is shown in Figure 2. Each green space had about 2 peak time points for exercise. The behavior maps of the green spaces at peak time points are shown in Table 9. The participants showed behavioral adaptability when exercised in the sunny and shelter sites; some of them changed their exercise positions with the changing sunlight and shadow. During the peak period 9:00–14:00, most of the sites in the park, the square, and the playground had sufficient sunshine, while most of the sites in the residential yard were in the shadow from the surrounding buildings (Table 10).

#### 3.3.3. Factors in Supporting Exercise Duration and Frequency 

**Figure 3:**
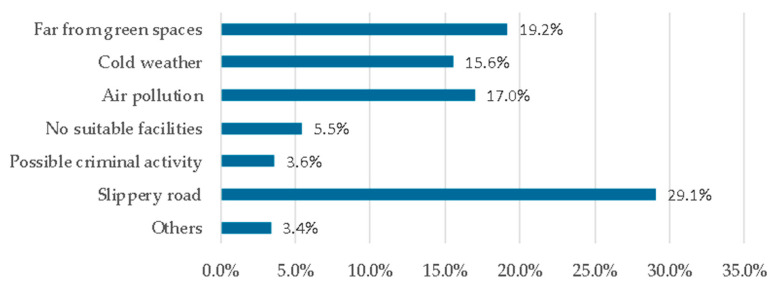
Factors hindering consistent exercise in the green spaces.

**Figure 4:**
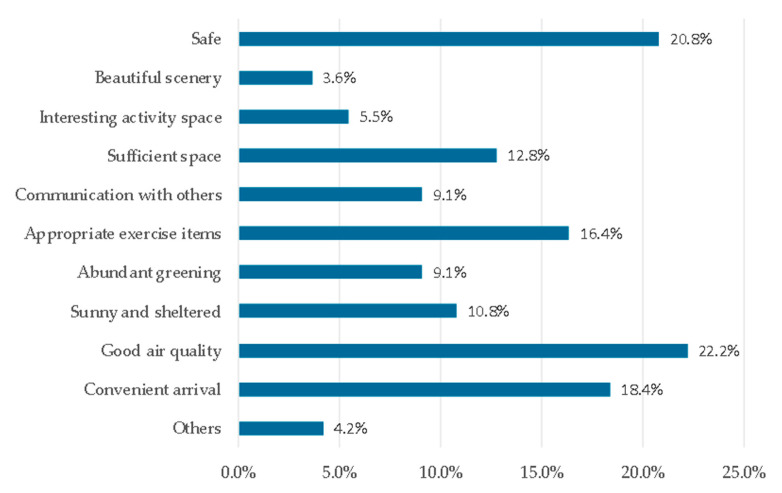
Factors promoting consistent exercise in the green spaces.

**Figure 5:**
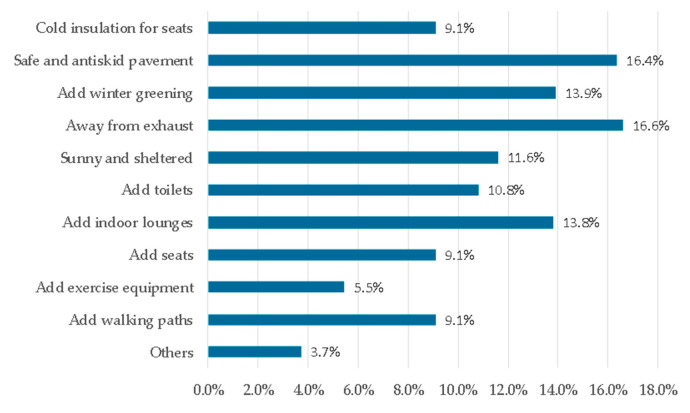
Demand factors for the green spaces.

According to the results of the questionnaire, the main factors that hindered seniors from exercising consistently in the green spaces were the slippery road (29.1%), bring far from green spaces (19.2%), air pollution (17.0%), and cold weather (15.6%) (Figure 3). The main factors that promoted exercise consistently in the green spaces were good air quality (22.2%), safety (20.8%), convenient arrival (18.4%), and suitable exercise items (16.4%) (Figure 4). The demand factors for the green spaces mainly included being away from exhaust (16.6%), access to a safe and anti-skid pavement (16.4%), adding winter greening (13.9%), and adding lockers and lounges (13.8%) (Figure 5).

According to the results of the interview with the patients, the main reasons the elderly patients with cardiovascular disease failed to exercise consistently in the green spaces were due to the insufficient safety measures and inaccessibility of the green spaces. The most frequently mentioned barriers to exercise engagement in green spaces by the participants were “slippery roads”, “medical accidents”, and being “far from the green spaces”. Some comments included: *I dare not go to exercise in the green spaces far from my home, the road is too slippery; I exercise almost every day, but I don’t go to exercise if it’s frozen; I only walk around my home, all know that the green space environment is good, but if I suddenly fall down, no one is around, what to do if there is an accident, my family cannot set their mind at rest.* In addition, stroke patients, whose physical mobility was weak, faced more obstacles and took a longer time to get to the green spaces (such as crossing the road): *we walk too slowly, when we cross the road slowly, the drivers would wait for and dislike us; it’s not convenient to go too far for exercise.* The results of interview with the doctors also showed that the most important factor of the green spaces was safety for the elderly who exercised there.

The field observation showed that the security threat of exercise in the green spaces mainly came from the sidewalks and sites with snow and ice. The clearing of snow in most of the sites was timely, but there were was snow and ice on the sidewalks connecting the green spaces, which became a potential risk for seniors when arriving at the green spaces. The pavement of some sites had insufficient skid resistance. Moreover, there were not any first aid facilities in the green spaces.

#### 3.3.4. General Factors of the Green Spaces 

When combining the exercise characteristics gleaned from the questionnaire, interview, and field observation results, the general factors of the green spaces in supporting exercise for cardiovascular health could be summarized as facilities allocation, comfort, safety, accessibility, and air quality. The images of the representative sampling sites reflecting these factors are shown in Table 11.

## 4. Discussion

The aim of this study was to identify the exercise characteristics of the elderly residents in the green spaces and compare them with the principles of the exercise prescription for cardiovascular health, in order to identify the deficient factors of the green spaces in supporting exercise for cardiovascular health, and to put forward optimization design implications, which could be conductive to increasing the potential benefits of the green spaces to cardiovascular health in the context of a winter city. There are a large number of elderly residents (a group with the highest risk of cardiovascular disease) in winter cities. Those of a higher socio-economic status can migrate to warmer areas during winter, while most of the middle- and low-income elderly are more dependent on daily health resources and face more health risk. Low-cost and daily accessible green spaces can play an important role in promoting cardiovascular health among these elderly residents. In the context of regional climate, both behavioral and spatial factors are greatly affected. In view of the specific health problems, it is meaningful to plan supportive green spaces factoring the exercise prescription and characteristics of this group, who are at high risk. 

### 4.1. Elaboration of the Factors and Design Implications

The results of our study show that: (1) at the behavioral level, some of the participants exercised inconsistently with the principles of the exercise prescription for cardiovascular health; about 42.4% of the participants had an insufficient total amount of exercise and about 10.6% of the participants exercised in the early morning; (2) at the spatial level, the deficient factors of the green spaces in supporting exercise for cardiovascular health mainly included facilities allocation, comfort, safety, accessibility, and air quality.

In terms of the exercise items, the elderly mostly chose low-intensity exercise according to their individual physical conditions, and these items were consistent with the prescription principles and conducive to cardiovascular health. The green spaces were able to support most of the exercise items, but the facilities allocation still needed to be improved when it came to finer details. In terms of exercise time, the elderly who usually exercised in the early morning due to cognitive reasons, habit, and other factors were at risk in terms of cardiovascular health. In addition, there was still a gap in the comfort of the green spaces during the appropriate period of exercise. In terms of exercise duration and frequency, some elderly residents insisted on exercising regularly, while others were unable or unwilling to exercise regularly. The safety and accessibility of the green spaces, air quality, and cold weather were the main factors hindering consistent exercise. For the elderly patients with cardiovascular disease, safety and accessibility were particularly important. The next paragraphs elaborate on these factors and provide implications for the design of the green spaces.

Facilities allocation: Our study found that although the green spaces supported many types of exercise items beneficial to cardiovascular health, the elderly, especially the elderly participants with cardiovascular disease, had higher support demands for routine and rehabilitation exercise activities due to their weakness and low physical activity threshold. Our results are in line with the findings of studies that show the elderly have higher requirements for the walking conditions, rest facilities, and barrier free facilities in the green spaces due to their weak individual ability [62,63]. In combination with the specific exercise characteristics of the elderly in the study, the optimization measures can be taken with as follows. With regards to the elderly participants’ walking characteristics, the walking paths should be continuous, circular, and level in height. In addition, a special walking path can be set up for the elderly, and the fast and slow zones can be set on the walking paths. For example, the playground should provide double ring runways: the inner ring as fast walking area and the outer ring as a jogging area. Moreover, the rehabilitation training paths and facilities should be set and surrounded by handrails to protect patients and aid exercise. Meanwhile, rest seats should be set on sides of the exercise sites. Also, auxiliary facilities that can aid exercise should be installed, including railings for stretching, and facilities for hanging clothing items and other goods. Finally, he elderly need to be better informed about scientifically proven and safe exercise methods, so it is necessary to provide guidance facilities with instructions, including exercise prescription examples and principles.

Comfort: Our study found that in the winter, the elderly showed behavioral adaptability in finding shelter and sunny areas to exercise in, but there was still a gap in the comfort of the green spaces in terms of the appropriate period of activity. Studies have found that the thermal comfort of a green space is closely related to the participation rate [64,65]. A practical measure study in a winter city in China also found that small changes in outdoor temperature affects the number of users: in winter, for every 1.5 ℃ rise in the temperature of the square, the number of the users of the square increased by 10 [66]. Comfort not only affects the exercise participation rate, but also reduces the health risks caused by low temperatures. To create a comfortable exercise environment would thus be beneficial for protecting the cardiovascular health of the elderly as well as increasing their exercise participate rates. In response to the study findings, we advise the following measures to be taken. Sunlight conditions of the exercise areas should be guaranteed. In accordance with the sunshine analysis, the sites of the green spaces that have good sunshine conditions during the appropriate exercise period of the day should be selected as the exercise areas. In addition, an indoor or semi-indoor space for resting should be set, and a closed storage cabinet for storing clothes and shoes should be installed.

Safety: The safety of the green spaces is a general but important factor to consider for the elderly in winter cities. International studies have documented that fear of injury is a common barrier to exercise participation, especially among the elderly [67,68,69]. Different from the safety problems (such as public security) faced by people in other areas [70,71], the elderly, especially those with cardiovascular disease, were most worried about facing medical emergencies in the winter, especially accidents caused by slippery sidewalks and sites due to snow and ice. An attempt should be made to address these concerns. Slippery surface material should not be used in the pavements in the green spaces; carpet can be laid on the exiting slope or slippery surface pavement. In addition, a makeshift or temporary ceiling can be added on the exercise sites, and the street lamps on both sides of the path should not have any protruding designs where snow and ice could collect. In terms of medical accidents, emergency call devices should be set in the area near the exercise sites, so as to help the elderly get assistance quickly in the event of any accident or injury.

Accessibility: Our study found that the support of the green spaces for accessibility involved not only distance, but also the safety and convenience of the sidewalks, and the health status of the elderly themselves. This is in line with results from a study showing that the accessibility factors of the green spaces include not only proximity on the physical dimension, but also safety on the social dimension and health on the individual dimension, which affects the accessibility of the green spaces cumulatively [72]. Although it is an important strategy to arrange the green spaces near residential districts, the safety of the sidewalks affected by the severely cold climate as well as the weak physical ability of the elderly should be considered. To address these barriers, the following strategies could be considered. In combination with the commonly used GIS analysis method, a fairness analysis and supply–demand matching analysis of the green spaces should be carried out, based on the distribution of the green spaces and the elderly residents. Furthermore, the number of green spaces should be increased and the road network structure should be optimized in the areas with poor accessibility and a dense distribution of the elderly. Also, special attention should be paid to the safety of the sidewalks connecting the entrances of the green spaces and priority snow removal level of the urban sidewalks should be established [73]. In order to reduce the obstacles to the elderly patients’ movement when they cross the motorway, the strategy of extending the signal lamp duration at intersections to improve the crossing convenience of people with relatively weak mobility in the New York City’s Active Design Guidelines [74] is an effective approach that can be applied around the green spaces in China.

Air quality: Air quality was another factor that was found to affect the green spaces’ ability to support exercise. The air quality of a green space affects the concentration of particles in a certain range around it [75]. However, when deciduous trees and plants wither in winter in winter cities, the air purification capacity of the green space are weakened. An experimental study found that evergreen trees like pine and cypress in series play a role in air purification in the winter [76]. Another evidence-based study showed that the morphology and height of shrub were better than that of arbor and ground cover herbaceous vines in terms of particulate matter of human respiration height [77]. Therefore, the number of evergreen trees should be increased to reduce the atmospheric particulate matter in the green spaces in winter cities, and more shrubs should be planted in the exercise areas. At the same time, in view of the problem of air pollution in the road sections adjacent to the park, we suggest planting an evergreen tree green belt and avoid arranging equipment, walking paths, and other exercise sites there.

### 4.2. The Winter City Context 

A key feature of this study is that it took place in a winter city context. The factors that support exercise in the green spaces in winter cities are not identical to those in non-winter cities. For example, in Singapore, the high temperature is a barrier factor for exercise in the green spaces [78], which is quite different from winter city conditions. Meanwhile, in some European countries [79] and in North America [80], the cold weather is a barrier to outdoor exercise, which is similar to our results. Under the influence of a severely cold climate, both the elderly residents’ enthusiasm and the safety and comfort of the green spaces decreased. In view of the special exercise characteristics of the elderly, especially those with cardiovascular disease, the green space factors have different connotations related to temperature, sunshine, ice, and snow.

The green spaces in the winter cities have a foundational ability to support exercise for cardiovascular health, as per the prescription principles, but there are some areas where they fall short. Facilities allocation, comfort, safety, accessibility, and air quality still need to be optimized, and measures should be taken to bridge the gap, especially when considering the impact of the cold climate.

In addition, we should be alert to the risk of exercise for the elderly in the winter in view of the special regional health problem of cardiovascular disease. Measures could be taken at both behavioral and spatial levels [81]. For example, wearing warm clothing and hats and following scientific exercise guidance and principles at the behavioral level, and ensuring sunlight conditions during the exercise period, and setting up guidance facilities with instructions and emergency call devices in the green spaces at the spatial level may help to reduce the risk of accident or injury. This study mainly focuses on the spatial level and proposes targeted design implications. Currently, there are few design guidelines set in place involving the green space design for promoting health in winter cities in China. It is worth picking up and adopting relevant information on this topic from other countries, such as Vermont’s Addressing Exercise Accessibility in Winter Conditions [82], the New York City’s Active Design Guidelines [74], and Design a Healthy Los Angeles [83] in the U.S., and Winter Design Guidelines in Edmonton [84], Canada. In future studies, the factors and strategies at the behavioral level should be further explored, such as individual prescription recommendations and the collection and publication of guidelines for winter exercise methods. At the spatial level, the design guidelines of the green spaces in winter cities considering the special exercise characteristics of the elderly should be compiled. These guidelines can help to implement the specific factors that are difficult identify by quantitative evaluation indicators, but more related to the qualitative spatial attributes such as feelings of security, comfort, and so on. In general, in combination with the high incidence of heart health problems in China, green space planning and design in the winter cities should be given greater attention so as to implement better support structures for the elderly.

### 4.3. Strengths and Limitations 

The main strengths of this study include its focus on high-incidence health problems in the context of a winter city, its consideration of exercise characteristics of the elderly residents (a group at high risk of cardiovascular disease) and exercise prescription for cardiovascular health, and its supportive factor analysis of the green spaces. We managed to recruit a sample of elderly residents, the majority of which belonged to middle- and low-income groups. The elderly individuals with relatively low socio-economic status could not afford to migrate to the southern areas in winter, and are thus most in need of daily green space resources. In addition, this study combined mixed methods (questionnaire, interview, and field observation) to facilitate an in-depth understanding of exercise characteristics of the elderly and supportive factors of the green spaces. Further, we identified how the green space factors supporting exercise of the elderly residents in a winter city are consistent with or different from results in other studies. Importantly, our findings add new insights to the literature regarding the green spaces and cardiovascular health.

We acknowledge that this study has limitations. First, for the supportive factors of the green spaces, we mainly focused on subjective perception rather than quantitative measurement using the qualitative methods. In the context of the winter cities, the factors of the green spaces in supporting winter exercise of the elderly involved the space quality, subjective feelings, the movements and the behavioral adaptability of the elderly, which were difficult precisely quantify. Meanwhile, some studies have pointed out that subjective perception factors are more reflective of actual green space use than objective measurement factors [85,86]. In addition, qualitative research may not provide definitive answers to such complex questions, but it can yield a better understanding and provide a springboard for further focused work [47]. In future studies, the scientific measurement of the subjective perception factors needs to be further verified through a larger study population, making these factors generalizable. Secondly, some elderly participants had a weak understanding of the problem, while some liked to chat; the outdoor weather was cold, and long-term outdoor survey was a challenge for both the participants and interviewers, so the sample we obtained was only a small one of the elderly residents. Finally, there are many types of cardiovascular diseases, and there are certain differences in the prescriptions adopted for different diseases. The reference contents we adopted were the general principles of exercise prescription for cardiovascular health, and more detailed exercise requirements and methods as well as corresponding supportive factors of the green spaces need to be explored.

## 5. Conclusions

Although the mechanism of how the green spaces influence cardiovascular health involves social interaction, air environment, personal factors, and other various factors, exercise appears to be one critical mediating factor recognized by most studies. Taking the severely cold climate in winter cities into consideration, this study links the exercise characteristics of the elderly residents in the green spaces with the principles of exercise prescription for cardiovascular health, identifies the deficient factors of the green spaces in supporting exercise for cardiovascular health, and then puts forward targeted design implications. The findings are expected to provide clues for the green space planning and design in winter cities to support exercise for cardiovascular health, which may play an important complementary role for disease prevention and treatment and rehabilitation for the elderly and other patients with cardiovascular disease in winter cities. In addition, future research should integrate more intervention strategies involving individual level, and the measurement of the factors and more planning and design strategies need to be verified and further studied in larger samples.

## Figures and Tables

**Table 1:** Participant characteristics in formal questionnaire and interview survey.

Characteristics	Participants in Questionnaire	Participants in Interview
Number *n*	188	40
Gender *n* (%)		
Male	105 (55.9%)	22 (55.0%)
Female	93 (49.5%)	17 (43.0%)
Mean age, years (SD)	67.5 (6.7)	68.0 (5.9)
Education *n* (%)		Not assessed
primary school and below	30 (16.0%)	
junior high school	38 (20.0%)	
high school	68 (36.0%)	
universities and colleges	53 (28.0%)	
Master and above	0 (0%)	
Monthly income *n* (%)		
≤2000 yuan	58 (30.9%)	
2000–5000 yuan	90 (47.9%)	
5000–10,000 yuan	40 (21.2%)	
≥10,000 yuan	0 (0.0%)	

SD: standard deviation.

**Table 2:** Exercise items of the participants in the green spaces.

Exercise Items	*n* (%)
Jogging	21 (11.2%)
Walking	96 (51.2%)
Dancing	4 (2.3%)
Exercise with equipment	56 (30.0%)
Tai Chi / Baduanjin	5 (2.9%)
Skating	4 (2.3%)

**Table 3:** Exercise time of the participants in the green spaces.

Exercise Time	*n* (%)
6:00–8:00	20 (10.6%)
9:00–11:00	81 (43.1%)
12:00–14:00	43 (22.9%)
15:00–17:00	27 (14.4%)
18:00–20:00	17 (9.0%)

**Table 4:** Exercise time of the participants in different exercise duration groups in the green spaces.

Exercise Duration	Exercise Time *n* (%)				
	6:00–8:00	9:00–11:00	12:00–14:00	15:00–17:00	18:00–20:00
≤10 min	2 (1.1%)	5 (2.7%)	4 (2.1%)	1 (0.5%)	0 (0.0%)
10–20 min	1 (0.5%)	18 (9.6%)	1 (0.5%)	1 (0.5%)	3 (1.6%)
20–30 min	2 (1.1%)	16 (8.5%)	9 (4.8%)	7 (3.7%)	5 (2.7%)
30 min–1 h	4 (2.1%)	8 (4.3%)	5 (2.7%)	4 (2.1%)	1 (0.5%)
1–2 h	5 (2.7%)	18 (9.6%)	16 (8.5%)	9 (4.8%)	7 (3.7%)
2–3 h	4 (2.1%)	9 (4.8%)	3 (1.6%)	2 (1.1%)	0 (0.0%)
>3 h	2 (1.1%)	7 (3.7%)	5 (2.7%)	3 (1.6%)	1 (0.5%)

**Table 5:** Exercise duration and frequency of the participants in the green spaces.

Exercise Duration	*n* (%)
≤10 min	12 (6.4%)
10–20 min	24 (12.8%)
20–30 min	39 (20.7%)
30 min–1 h	22 (11.7%)
1–2 h	55 (29.3%)
2–3 h	18 (9.6%)
>3 h	18 (9.6%)
Exercise frequency	n (%)
≤3 days	54 (28.5%)
3–5 days	20 (10.8%)
5–7 days	114 (60.7%)

**Table 6:** Mean exercise frequency of the participants in different exercise duration groups in the green spaces.

Exercise Duration	Mean Exercise Frequency
≤10 min	4.3 days
10–20 min	4.8 days
20–30 min	4.9 days
30 min–1 h	5.3 days
1–2 h	6.1 days
2–3 h	6.0 days
>3 h	7.0 days

**Table 7:** The area of the green spaces.

Green Spaces	Park	Square	Playground	Residential Yard
Area	0.86 hectare	0.61 hectare	0.37 hectare	0.18 hectare

**Table 8:** Exercise item support of different spatial forms of green spaces.

Exercise Items	Green Spaces
Walking	The path in the park, the edge of the square, the pathway of the playground, the path in the residential yard
Exercise with equipment	The area with exercise equipment in the park, the square, the playground and the residential yard
Dancing	The square, the vacant lands in the park, the playground and the residential yard
Stretching	The area with railings and exercise equipment and other things by which people can do stretching exercise in the park, the square, the playground, and the residential yard
Tai Chi	The square, the vacant lands in the park and the residential yard

**Table 9:** Exercise behavior maps of the green spaces at the peak time points.

Green Spaces	Exercise Behavior Maps	
Park	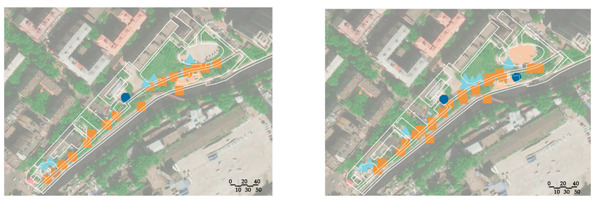	
	10:00	19: 00
Square	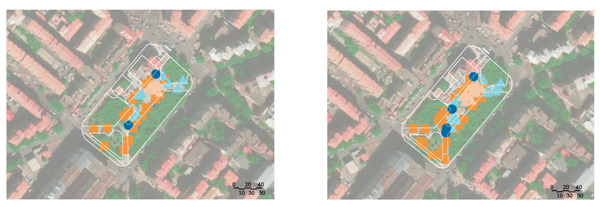	
	10:00	14:00
Playground	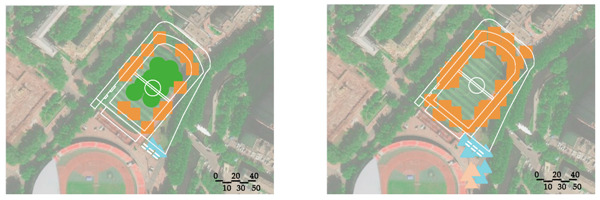	
	11:00	19:00
Residentialyard	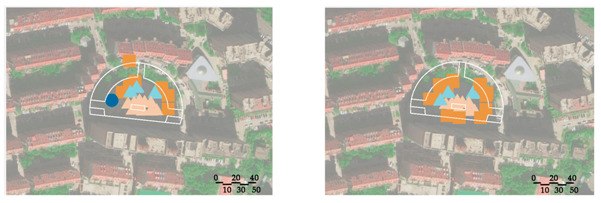	
	14:00	19:00
Legend	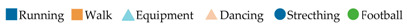	

**Table 10:** The sunlight conditions of the observation sampling sites during the peak period.

Sampling Sites		9:00	10:00	11:00	12:00	13:00	14:00
Park	P1	1	0	0	1	1	1
	P2	0	1	1	1	1	1
	P3	1	1	1	1	1	1
	P4	1	1	1	1	1	1
	P5	1	1	1	1	1	1
	P6	1	1	1	1	1	1
Square	S1	0	0	1	1	1	1
	S2	1	1	1	1	1	1
	S3	1	1	1	1	1	1
	S4	0	1	1	1	1	1
	S5	0	0	1	1	1	1
	S6	0	1	1	1	1	1
	S7	0	0	1	1	1	1
	S8	0	0	1	1	1	1
Playground	PL1	1	1	1	1	1	1
	PL2	1	1	1	1	1	0
	PL3	1	1	1	1	1	1
	PL4	1	1	1	1	1	1
Residential yard	R1	0	0	0	1	1	0
	R2	0	0	0	0	1	1
	R3	0	0	0	0	1	1
	R4	0	0	0	0	1	1

Note: P represents the park, S represents the square, PL represents the playground and R represents the residential yard.

**Table 11:** The images of the representative sampling sites reflecting the general supportive factors of the green spaces.

Spatial Factors	The Images of the Representative Sampling Sites		
Facilities allocation	Sites	P2, S6, PL2, R3	P1, P3, P5, P6, P7, S2, S7, S8, PL4, R3, R4
	Images	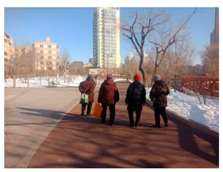	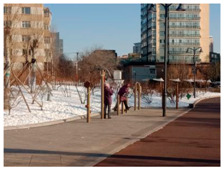
	Description	The participants preferred walking on flat, continuous, and connected paths.	There were no hanging facilities for articles carried by the participants.
Comfort	Sites	R1, R2, R3, R4, R5, R6, R7, S3, S4, S6, PL2, PL4	P1, P6, PL1, S6, R2
	Images	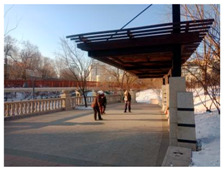	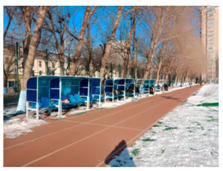
	Description	The participants showed behavioral adaptability when choosing the sunny and shelter sites to exercise.	The clothes and shoes of the participants were stored exposed to the cold air.
Safety	Sites	R1	P7, R4
	Images	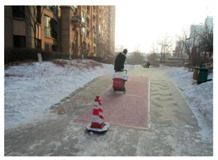	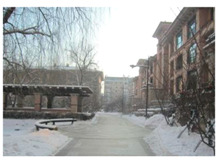
	Description	The carpet was laid on a slope by the property personnel.	Snow fell repeatedly from the top of the protruding lamp post.
Accessibility	Sites	P3, PL4	R2
	Images	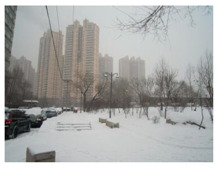	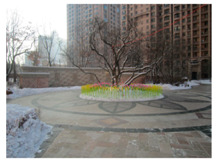
	Description	The snow on the sidewalks connecting the green spaces was not cleared in time.	The residential yard had an advantage in accessibility.
Air quality	Sites	P4, P6, PL4	P1, S6, PL2
	Images	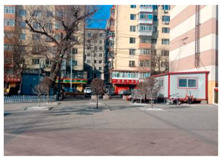	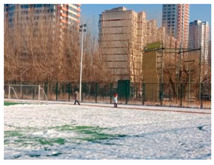
	Description	The vegetation withered and the automobile exhaust gas isolation was insufficient.	Due to the isolated green belt, direct contact with automobile exhaust was avoided.

## Appendix A

**Table A1:** The classification standards for different income groups from the National Bureau of Statistics of China.

Monthly Income	Income Groups
≤2000 yuan	The low-income group
2000–5000 yuan	The middle-income group
5000–10,000 yuan	The middle-high-income group
≥10,000 yuan	The high-income group
